# Many Interesting Paragraphs

**Published:** 1894-08

**Authors:** 


### Medical News and Miscellany.

**Dr. E. Cross,** so long resident in San Antonio, where he successfully conducted a large gynecological infirmary, has removed to Monterey, Mexico.

**Married,** at Taylor, Texas, July 24th, ult., Miss Annie Doak, daughter of Dr. A. V. Doak, to Mr. E. R. Adams, all of Taylor.

---

**For Sale.**—My home and good will. A great bargain. Fine location, in Henrietta, Texas. Address:

S. G. BITTICK, M. D., Henrietta, Texas.

---

**Dr. Wm. Osborn** has removed from Waco to Rockdale, and we are pleased to learn has begun what promises to be a prosperous career. He has our best wishes for success, and he certainly deserves it.

---

**Club.**—Texas Medical Journal........................$2 00
Literary Digest (Weekly).................... 3 00

We will send both journals to any person not already a subscriber to the last-named publication, for $4.40.

---

**Colorado County Medical Society.**—A letter from Dr. Sam B. McLeary, of Weimar, says: "We have organized a County Medical Society here in Colorado county, and would like to have a copy of the by-laws of the Austin District Medical Society by which to frame ours. We would also be glad to report our proceedings in the TEXAS MEDICAL JOURNAL." (No proceedings received to date--ED.)

---

**Medical Examining Board.**—Hon. F. R. Morris, Judge of the 26th Judicial District Court, has appointed Drs. J. W. McLaughlin, T. J. Bennett and S. E. Hudson the Board of District Medical Examiners for the 26th District. The Board organized by electing Dr. McLaughlin, President, and Dr. Bennett, Secretary. The Board will meet at the office of Drs. Bennett & Hudson quarterly, on the first Wednesday of January, April, July and October of each year.

---

**General Dabney H. Maury's** book, "Recollections of a Virginian in the Mexican, Indian and Civil Wars," just issued in splendid style by the Scribners,—a most charming book,—will be sent post-paid to any of our readers in club with the TEXAS MEDICAL JOURNAL for $3.02 for the two. (The price of the book alone, including postage, is $1.62.) This book is the "latest," and is all the talk; everybody wants to read it. Gen. Dabney's daughter, Mrs. Rose M. Pollard, Houston, Texas, is the general agent. Subscriptions at above club rates must be sent to this office; full rate subscriptions to Mrs. Pollard. In our next issue the work will be reviewed; meantime hurry up your orders as this, the second edition, will not last long; it sells like "hot cakes." Save 50c. by clubbing.

---

**Marlin's Artesian Well**—A spouting hot well: Dr. S. P. Rice, of Marlin, one of the JOURNAL's best friends, sends us a "Souvenir" (along with renewal of subscription for the tenth year) in the shape of a neat pamphlet descriptive of this great and wonderful well, bored in the town of Marlin, Falls county. It is a curiosity, as well as a valuable therapeutic agent, the temperature of the water being 144° F., the hottest artesian well water in the United States. The well is 3350 feet deep, and spouts with a pressure of 98 pounds per square inch. It is a hand-made Geyser that rivals in beauty and grandeur the famous Giant Geyser of the Yellowstone region. The good people of Marlin should be able to turn this bonanza to profitable use in more ways than one, which suggest themselves; while the learning, skill and enterprise of her physicians, Nettles, Rice and Dupree, should be utilized to make the town a Mecca for invalids. If Marlin has not a fine hotel, and other accessories of a first-class health resort, within a short time, and this valuable "find" put to some use, it will not be because her medical men fail to appreciate it. It is a big bid for a safe investment for capital. Write to Dr. Rice or Nettles for a description of this wonderful well.

---

**Acknowledgements.**—The JOURNAL makes its most grateful (and graceful) acknowledgements for the very prompt and courteous manner in which a large number of our subscribers responded to our little "call," on page 33 of last month. The remittances came pouring in, arrears and advance payments alike, in liberal numbers and amounts, each one accompanied by pleasant words; words of cheer and encouragement, which are to us as refreshing as the showers that fall on the parched and dusty highways. There is a deal of pleasure in the relation of editor and subscriber under such conditions, and the kind appreciation of our efforts expressed by so many, and in such nice terms, gives us renewed energy and strength, new determination to still make the Red-back the *best in the land*, and it gives us, at the same time, the means to do it! We return many thanks; and feeling easy now, on the score of Betty and the Baby's having shoes and stockings this winter, we can "command our mind" and apply it to the interests of the Red-back and its backers.

INTERESTING AND IRREPRESSIBLE.—"Accept our sympathy,' says an old standby, "for your being financially so near the bottom of your pocket." "I take pleasure in remitting you $2.00 for your interesting and irrepressible JOURNAL for the ensuing year, wishing you abundant success and pleasure, and all the lucre necessary for your future plans and happiness."

Another says, "I dearly love the JOURNAL; full of sound, sensible reading for all."

The *Fayette County Paper*, edited by a leading physician, says:

The TEXAS MEDICAL JOURNAL for July is to hand, and with this number enters upon its tenth year of existence. Its success has been phenomenal in medical journalism, and without straining the English, we can say that it easily stands at the head of the list. This number is especially commendable, from the fact of the able opening paper by our popular State Health Officer, Dr. Swearingen, on "Spinal Concussion," and the editorial on the same subject by Dr. Daniel. This subject is likely now to get another pretty thorough ventilating. This number of the JOURNAL contains other valuable papers which all intelligent Texas physicians will want to read. We especially commend the paper on "Malarial Hæmatinuria," by T. F. Oates, M. D., of Mexia, Texas. Austin, Texas, $2.00 a year.

Another says, "Can't do without it, stringency or no stringency."

Another, still, says: "I'll cut off my rations of tobacco rather than do without the JOURNAL. Here goes for 1895; and good luck to you."

And so on; we could (and would like to) reproduce scores of such kindly expressions.

